# Chronic oxycodone induces axonal degeneration in rat brain

**DOI:** 10.1186/s12868-018-0417-0

**Published:** 2018-03-23

**Authors:** Ruping Fan, Lisa M. Schrott, Thomas Arnold, Stephen Snelling, Meghana Rao, Derrel Graham, Angela Cornelius, Nadejda L. Korneeva

**Affiliations:** 1 Department of Emergency Medicine, Louisiana State University Health Sciences Center, 1501 Kings Highway, Shreveport, USA; 2 Department of Pharmacology, Toxicology, and Neuroscience, Louisiana State University Health Sciences Center, 1501 Kings Highway, Shreveport, USA

**Keywords:** Axon, Myelin basic protein, Oxycodone, Morphine, Opioid, Nucleus accumbens, Cortex, Cerebellum, Brain stem, Oxidative stress, Integrated stress response, Phosphorylated eIF2α, Translation, ATF4, Bax, XIAP, MCF7, ISRIB

## Abstract

**Background:**

Chronic opioid therapy for non-malignant pain conditions has significantly increased over the last 15 years. Recently, the correlation between opioid analgesics and alternations in brain structure, such as leukoencephalopathy, axon demyelination, and white matter lesions, has been demonstrated in patients with a history of long-term use of prescription opioids. The exact mechanisms underlying the neurotoxic effect of opioids on the central nervous system are still not fully understood. We investigated the effect of chronic opioids using an animal model in which female rats were orally gavaged with 15 mg/kg of oxycodone every 24 h for 30 days. In addition we tested oxycodone, morphine and DAMGO in breast adenocarcinoma MCF7 cells, which are known to express the μ-opioid receptor.

**Results:**

We observed several changes in the white matter of animals treated with oxycodone: deformation of axonal tracks, reduction in size of axonal fascicles, loss of myelin basic protein and accumulation of amyloid precursor protein beta (β-APP), suggesting axonal damages by chronic oxycodone. Moreover, we demonstrated activation of pro-apoptotic machinery amid suppression of anti-apoptotic signaling in axonal tracks that correlated with activation of biomarkers of the integrated stress response (ISR) in these structures after oxycodone exposure. Using MCF7 cells, we observed induction of the ISR and pro-apoptotic signaling after opioid treatment. We showed that the ISR inhibitor, ISRIB, suppresses opioid-induced Bax and CHOP expression in MCF7 cells.

**Conclusions:**

Altogether, our data suggest that chronic opioid administration may cause neuronal degeneration by activation of the integrated stress response leading to induction of apoptotic signaling in neurons and also by promoting demyelination in CNS.

## Background

In 1999, pain was introduced as a fifth vital sign along with body temperature, pulse, respiration, and blood pressure [1]. Since then, in parallel with dramatic increase in opioid prescriptions, the rate of admission of treatment-seeking patients for opioid addiction and opioid-overdose death nearly quadrupled in 2010 [2]. Recently, concern regarding the effect of chronic opioid exposure on neuronal degeneration has emerged. A toxic effect of opioids on the central nervous system (CNS) has been described in a growing number of studies. Leukoencephalopathy, axon demyelination, and lesions in white matter have been documented mostly for heroin abusers [3–14]. It was speculated that the contamination in street-heroin or combustion by-products are responsible for its neurotoxic effect. However, similar lesions in white matter have been documented for methadone over-dosed patients [15–17], and recently was detected in a patient after morphine overdose [18] and a patient after acute oxycodone overdose [19], suggesting that leukoencephalopathy has a direct link to the toxic effects of opioid abuse. In 2010, a study investigating brain structures in a highly enriched group of prescription opioid-dependent patients demonstrated significantly decreased anisotropy in the axonal pathways in various brain regions responsible for impulse control, reward and motivation [20]. Interestingly, in this study, a decrease in white matter pathways has been demonstrated in every one of ten prescription opioid-dependent patients [20] suggesting that opioid-induced leukoencephalopathy is not a rare event but a general slowly progressing process leading to neuronal degeneration.

Interpretation of a study involving human subjects may be complicated by the possibility that patients have a genetic predisposition or lived in a certain environment that contributed to neurodegeneration and that prolonged administration of opioids is not a primary cause of leukoencephalopathy but an exuberant of individual patient factors. Animal models lack such complications. There are several animal studies that addressed the question whether opioid abuse triggers neuronal degeneration. Heroin-addicted rats demonstrated neuronal cell death in the cerebellum [21]. Rats treated with morphine expressed cognitive deficit [22] and demonstrated apoptosis in neuronal cells [23, 24]. However, there are no reports regarding the opioid-induced pathology in white matter in an animal model. To investigate the effect of chronic opioid administration on brain structures, we treated rats with either water or oxycodone (15 mg/kg) for 30 days and analysed changes in axons and also induction of the pro-apoptotic markers in animal brains. In our animal model we observed reduction in size of axonal bundles and deformation of axonal tracks. Moreover, oxycodone-treated animals demonstrated a decrease in myelin basic protein (MBP) level but an increase in amyloid precursor protein beta (β-APP) suggesting demyelination and axonal damage after chronic oxycodone exposure. We also observed increased levels of pro-apoptotic Bax and activated caspase 3 in oxycodone rat brain areas containing white matter.

Recently we showed that chronic oxycodone administration activates the integrated stress response (ISR) in rat brain [25]. The ISR triggers a general adaption mechanism that provides necessary support for cells to survive during stress and also promotes recovery. The key event in the ISR, regardless of the trigger, is phosphorylation of eIF2α that reduces general translation but allows ribosomal subunit to initiate translation of specific mRNAs containing upstream open-reading frames (uORF) in their 5′UTR via leaky scanning [26], particularly ATF4 [27]. ATF4 is a transcription factor that controls expression of genes involved in protein synthesis, redox status and autophagy [28]. However, prolong activation of the ISR may lead to exhaustion of cellular supplies and trigger activation of apoptosis [29, 30]. Using breast adenocarcinoma MCF7 cells, which are known to express the opioid receptors, we observed induction of the ISR and pro-apoptotic signaling after opioid treatment, which were suppressed by the ISR inhibitor, ISRIB.

## Methods

### Animal model

Female 60 day-old Sprague–Dawley rats (180–240 g) were purchased from Harlan Indianapolis, IN. They were fed chow and water ad libitum and maintained on a 12-h light/dark cycle. The animals were housed three to a cage and allowed to acclimate for at least 1 week before experiments were conducted. The protocol for animal studies was approved by the Louisiana State University Health Science Center, Institutional Animal Care and Use Committee. Animals were treated as it is described in [25]. Rats were assigned to one of two groups (*n *= 3/group) administered either oxycodone (Mallinckrodt Inc., St. Louis MO) or its vehicle water. Oxycodone (15 mg/kg) Fisher’s PLSD or water was administered by oral gavage (volume of 1.0 ml/kg) every 24 h for 30 days. The oxycodone-treated group was compared directly to the water-treated control group, which was handled, treated, and sacrificed at the same time and under the same conditions. For statistical analysis this treatment [three water-(W) and six oxycodone-treated (O1 and O2) animals] was repeated at least three times. To determine the toxicity of the oxycodone, rats were weighed daily. There was no significant weight loss in either group of animals. In our earlier study, we compared the antinoceptive effect of 7.5 and 15 mg/kg oxycodone and the development of tolerance in rats using a hot plate assay [25]. We demonstrated that high dose, 15 mg/kg, oxycodone treatment led to the development of effective chronic analgesia, similar to that seen in human dosing. It was shown that oral doses of 16 mg/kg/day of oxycodone in rats are equivalent to approximately 2.6 times an adult human dose of 60 mg/day [31]. The initial dose to provide analgesia in humans is 5–15 mg orally every 4–6 h. The maximum human daily dosage may reach more than 200 mg for patients experiencing breakthrough pain [32]. Thus, oral treatment of rats with 15 mg/kg oxycodone daily is within the high dose range of human equivalent.

### Brain lysates preparation

For Western blot, rats in oxycodone-or water-treated groups were sacrificed by CO_2_ asphyxiation and decapitated 2 h after administration of the last dose. The brain lysate preparation has been previously described in [25]. Whole-brain tissues were harvested, and then tissues containing cortex, nucleus accumbens and cerebellum were collected in separate tubes containing ice-cold buffer A [50 mM Tris–HCl, pH 8.0, 140 mM NaCl, 5 mM KCl, and 6 mM MgCl_2_] substituted with 1 mM Na-vanadate. The corresponding tissues from three rats were pooled together in order to obtain enough material for all analyses. Tissues were homogenized in ice-cold buffer B [100 mM Tris–HCl, pH 8.0; 150 mM KCl, 10 mM MgCl_2_, 200 mM Sucrose, 5 mM DTT, 0.5 mg/ml heparin, 0.5% Triton™ X-100, 0.5% NP-40, and 0,5% deoxyholate, Complete™ EDTA-free protease inhibitor cocktail (Roche), and phosphatase inhibitor cocktails (Pierce Biotechnology, cat# 78420)], by 30 strokes with a Teflon/glass homogenizer, incubated on ice for 10 min, pipetted 10 times with 1 ml tip and centrifuged at 10,000×*g* at 4 °C for 5 min. The supernatants were collected and concentration of proteins in the supernatant was measured with Micro BCA™ Protein Assay Kit (Thermo Scientific, Pierce). Brain tissue lysates were stored at − 80 °C until further processing. We choose to investigate these brain areas based on their functions that may contribute to pain perception and also cognitive and motor impairment (cortex); to reward and the abuse potential (nucleus accumbens); and to mediation of respiratory depression and some of the ant-nociceptive and pro-emetic effects (brain stem). Striatum contains efferent and afferent pathways of the amygdala, which were shown to be affected in brains of the prescription-drug abusers [20].

### MCF7 cell culture and drug treatment

The MCF7 human breast adenocarcinoma cells (ATCC) were maintained in the Dulbecco’s modified Eagle’s medium (DMEM)/low glucose (Hyclone, Logan, UT) supplemented with 10% fetal bovine serum (Atlanta Biologicals Inc., Flowery Branch, GA) and 1% penicillin–streptomycin (Gibco, Carlsbad, CA). Cell treatment and harvest have been performed as previously described in [25] with some modifications. Cells were plated into 60 mm plates at 4–5 × 10^5^ cells per plate and allowed to adhere overnight. 2 h prior to drug treatment, cells were washed with warm PBS and supplemented with fresh media. In experiments with addition of the opioid-receptors antagonists, cells were first pre-incubated with either vehicle or 10 µM of naloxone hydrochloride (the pan opioid-receptor antagonists, Sigma), 0.3 µM of CTAP (the MOP receptor selective antagonist, Cayman Chemical) or 0.3 µM of naltridole (the delta-opioid receptor selective antagonist, Cayman Chemical) for 15 min. After that 10 μM oxycodone, morphine, DAMGO or equal volume of vehicle (0.1% DMSO in water final concentration) were added to the plate. Cells were allowed to grow for the next 24 h and then harvested. In experiments with addition of the ISRIB (Trans-ISRIB, Cayman Chemical), cells were grown in the presence of vehicle or 10 µM oxycodone, morphine or DAMGO and with or without of 40 nM ISRIB for 24 h. Each experiment was repeated at least three times using different batch of cell culture. For the MTT assay and immunofluorescent analysis of CHOP expression, MCF7 cells were incubated with vehicle, 2.5 µM or 25 µM DAMGO for 48 h.

### MCF7 cell lysates preparation

To analyze the expression of proteins, cells were lysed directly on a plate in 100 μl cold RIPA–EDTA buffer [50 mmol/L Tris–HCl, 150 mmol/L NaCl, 0.5% NP-40, 0.5% sodium deoxycholate, 0.5% Triton™ X-100, 0.1% SDS, and 5 mmol/L EDTA, pH 7.4] containing Phosphatase Inhibitor Cocktails 2 and 3 (Sigma, P5726 and P0044), and protease inhibitor (Mammalian ProteaseArrest™, GBiosciences, A Geno Technology Inc.). Cells were incubated on ice for 5 min, scraped, transferred to the Eppendorf, pipetted 10 times with 200 μl tip and centrifuged at 10,000×*g* at 4 °C for 5 min. The supernatants were collected and then protein concentration was determined using Micro BCA™ Protein Assay Kit (Thermo Scientific, Pierce).

### Western blot analysis

#### Brain tissue lysates

To analyse the expression of proteins in brain tissues, equal amounts of total protein were loaded on a 12% or 4–12% NuPAGE^®^ Novex^®^ Bis–Tris Gel (Invitrogen). The Full-Range Rainbow protein molecular weight marker (GE Healthcare Life Science) was loaded on the same gel to identify the position of specific proteins. Proteins were separated by SDS-PAGE gel and then transferred to a Nitrocellulose membrane (Bio-Rad) using a Mini Trans-Blot cell (Bio-Rad). Expression of specific proteins were determined by probing the membrane with antibodies against actin (dilution 1:8000; Sigma, A2066); APP (6E10, dilution 1:1000; Covance, SIG-39320); ATF4 (dilution 1:2000; Thermo Scientific, PA5-19521); Bax (dilution 1:2000; Cell Signaling, 2772s); GAPDH (dilution 1:8000; Fitzgerald, 10R-G109A); MBP (dilution 1:1000; Santa Cruz, sc-13914); NF (Neurofilament-L C28E10, dilution 1: 4000; Cell Signaling, 2837); tau (dilution 1:1000; Cell Signaling, 4019); phospho-tau (dilution 1:1000; Cell Signaling, 5383); XIAP (dilution 1:3000; R&D Systems, AF8221). The membranes were incubated with primary antibodies in 5% BSA in buffer TBS-T [20 mM Tris–HCl, 150 mM NaCl, and 0.1% Tween^®^ 20, pH 7.5] overnight at 4 °C, washed three times for 15 min with TBS-T, and incubated for 1 h at room temperature (RT) with anti-mouse secondary antibodies (dilution 1:20,000) for GAPDH and anti-rabbit secondary antibodies (dilution 1:20,000) for all other primary antibodies conjugated with horse-peroxidase (Vector Laboratories, Inc.) in 5% non-fat dry milk in TBS-T. Blots were developed with the Western Lightning ECL Pro development kit (PerkinElmer) and exposed to HyBlot CL autoradiography film (Denville Scientific). Quantitative analysis of Western blot images was performed using the ImageQuant TL software (GE Healthcare Life Science).

In experiments involving brain lysates, each sample contained the corresponding brain tissue from three rats. To analyze the effect of each treatment on the expression of proteins, β-APP, MBP, NF and XIAP signals were normalized to actin, and phospho-tau signal was normalized to total tau. The oxycodone data (O1 or O2) were then normalized to the corresponding water-control data (W) from the same treatment experiment. Results are presented as the mean of at least three independent treatment (drug administration) experiments ± SEM. To determine statistical significance of the effect caused by the treatment, data from oxycodone were compared to the water-control using Student’s t-test, and data with p value lower than 0.05 were considered to be statistically different.

#### MCF7 cell lysates

Ten micrograms of total protein of the MCF7 cell lysates were resolved on the 4–12% NuPAGE^®^ Novex^®^ Bis–Tris Gel (Invitrogen) by SDS-PAGE, transferred to a Nitrocellulose membrane (Bio-Rad) and then analyzed by western blotting as it is described above. Expression of specific proteins was determined by probing the membrane with primary antibodies against actin; ATF4; Bax (dilution 1:20,000; Proteintech Group, 50599-2-Ig) and anti-rabbit secondary antibodies conjugated with horse-peroxidase (dilution 1:20,000). Blots were developed with the Western Lightning ECL Pro development kit (PerkinElmer) and exposed to HyBlot CL autoradiography film (Denville Scientific). Quantitative analysis of Western blot images was performed using the ImageQuant TL software (GE Healthcare Life Science). ATF4 and Bax signals were normalized to actin in the same sample. The opioids data were then normalized to the corresponding vehicle data from the same experiment. Results are presented as the mean of three independent cell culture experiments ± SEM. To determine statistical significance of the effect caused by the treatment, data from opioids were compared to the vehicle, and data from opioids in the presence of naloxone were compared to the corresponding opioid alone, using Student’s t-test. Data from opioid in the presence of CTAP or naltridole were compared to the corresponding opioid alone using one-way ANOVA followed by Tuckey analysis. Data with p value lower than 0.05 were considered to be statistically different.

### Immunohistochemical analysis

For immunohistochemical analyses, three rats treated with water and three rats treated with oxycodone were anesthetized with injection of 65 mg/kg i.p. of sodium pentobarbital and then perfused through the aortic arch with 100 ml ice-cold saline followed by 400 ml ice cold 4% paraformaldehyde in 0.1 M sodium phosphate buffer, pH 7.4 (PB) as it is described in [25]. The whole brain was removed and placed in 4% paraformaldehyde in PB overnight at 4 °C, transferred to a 15% sucrose in 0.1 M PB for 24 h, then to 30% sucrose in PB for 24 h and then stored in 70% ethanol at 4 °C until further processing. Rat brain sections containing cortex and nucleus accumbens (plates 12–30, Rat Brain Atlas, Paxinos and Watson) or cerebellum (plates 122 and later, Rat Brain Atlas, Paxinos and Watson) were embedded in paraffin according to a standard protocol, cut into 10 μm thick slices and then mounted on glass slides, two or three consecutive slices on one slide (Millennia 1000). Tissue sections were deparaffinized by warming in the oven at 65 °C for 1 h, rehydrated in xylene, 100 and 95% solutions of ethanol and then in water. The antigens were retrieved by incubation of slides in sub-boiling 10 mM sodium citrate, pH 6.0 for 30 min. To reduce non-specific background sections were incubated with 3% hydrogen peroxide for 10 min at RT. Then each slide containing two or three sections of rat brain was incubated with two different dilutions of primary antibodies and no primary antibodies as a negative control. Primary antibodies against: APP (6E10, dilution 1:100); ATF4 (dilution 1:100); Bax (dilution 1:200; Cell Signaling, 2772s); cleaved caspase 3 (dilution 1:100; Cell Signaling, 9664); P-eIF2α (dilution 1: 100; Cell Signaling, 3398); NF (dilution 1:100); α-Synuclein (dilution 1: 2000; Cell Signaling, 3398); XIAP (dilution 1:100) were diluted in SignalStain Antibody diluent (Cell Signaling, 8112S). Sections were processed with either VectaStain ABC (to detect rabbit primary antibodies) or ImmPRESS Anti-Mouse Ig Rat adsorbed Polymer Detection Kits (to detect mouse primary antibodies) (Vector Laboratories, Inc.). The signal was visualized by the peroxidase substrate kit DAB (Vector Laboratories, Inc.). The nuclei were stained using the hematoxylin Gill’s 3 formulation (VWR) followed by wash in 0.2% ammonia water. Of note, that the slides stained with antibodies against cleaved caspase 3 were not counterstained with hematoxylin. The slides were then treated with graded alcohols and xylene. The coverslips were placed using HistoChoice^®^ Mounting Media (AMRESCO Inc.). Images were taken using light microscope BX43F (Olympus).

To analyse the bundle density in striatum: immunoreactivity signal was measured by ImageQuant TL software (GE Healthcare Life Science) and then “value” of the signal was divided to the “area” of the image. Data presented as relative density measured as the ratio of the mean values of intensities obtained from oxycodone exposed tissue to the bundle intensities in striatum of water-exposed rat brains. Results are presented as mean of at least three independent experiments using brains slides from three different animals (± SEM). To determine statistical significance, data were analyzed by Student’s t-test, and data with p value lower than 0.05 was considered to be statistically different. Analysis of cells in striatum presented as a percent of positively stained cells relative to total number of cells in the field. Three to four slides from each animal were analysed by 20X objective on a light microscope BX43F (Olympus). Data obtained from three animals for each treatment (400–500 total number of cells for each treatment). Brown-stained cells were count as positive. Data expressed as mean value of the percent of positive cells (± SEM). Statistical analysis of positively stained cells in oxycodone relative to water exposed striatum was performed using Student’s t-test and data with p value lower than 0.05 was considered to be statistically different.

### TUNEL analysis

Cell apoptosis was monitored by TUNEL detection kit (NeuroTACS II in Situ Apoptosis Detection Kit, Trevigen, R&D) according to manufacture protocol. Slides containing 10 μm thick slices of rat brain (as it is described in the “Immunohistochemical analysis” section above) were deparaffinized by warming in the oven at 60 °C for 5 min, rehydrated in xylene, 100, 95 and 75% solutions of ethanol and then in PBS. The tissues were permeabilized by incubation with NeuroPore solution™ for 27 min at RT. Then slides were immersed in quenching solution for 5 min at RT. After wash with TdT labelling buffer™, each tissue was incubated with 50 μl labelling reaction mixture for 1 h at 37 °C in humidified chamber. After wash with TdT stop solution™, slides were incubated with Streptavidin-HRP solution for 10 min at RT in humidified chamber, washed with PBS and immersed in DAB solution for 5 min at RT. Slides were counterstained with Blue Counterstain for 1 min and blued with ammonium water for 1 min. After wash in running tap water, tissues were dehydrated in graded alcohol and cleared in Xylene. A 50 μl drop of HistoChoice^®^ Mounting Media (AMRESCO Inc.) followed by a cover slip was placed on each brain tissue. The positive control slides containing water-or oxycodone-treated brain sections were pre-treated with TACS-Nuclease solution™ for 30 min at RT in humidified chamber followed by incubation with labelling reaction. Cells with brown staining in nucleus were considered as TUNEL-positive cells. Images were taken using light microscope BX43F (Olympus). Cells from three to five fields for each brain region has been investigated. The results are presented as a percent of TUNEL-positive cells in rat brain tissues exposed to water (W) oxycodone (O) and in control slides. Data presented as mean value of TUNEL-positive cells (± SEM). To determine statistical significance, data was analyzed by Student’s t-test, and data with p value lower than 0.05 was considered to be statistically different.

### Immunofluorescent analysis

#### Brain samples

Slides containing 10 μm thick slices of rat brain (preparation is described in the “Immunohistochemical analysis” section above) were deparaffinized by warming in the oven at 65 °C for 1 h, rehydrated in xylene, 100 and 95% solutions of ethanol and then in water. The antigens were retrieved by incubation of slides in sub-boiling 10 mM sodium citrate, pH 6.0 for 30 min and then cooled at RT for 20 min. After three times wash with PBS, cells were permeabilized by incubation in ice cold methanol for 5 min on a rocking platform. To reduce non-specific background sections were incubated with 3% hydrogen peroxide for 10 min at RT and then with 2.5% normal house serum 1 h at RT. Then each slide containing two or three sections of rat brain was incubated with two different dilutions of primary antibodies and no primary antibodies as a negative control. Primary antibodies against: GFAP conjugated to Alexa Fluor 594 (dilution 1:50, Cell Signaling, 8152), MBP (goat, dilution 1:400; Santa Cruz, 13914), and NF (rabbit, dilution 1:100) were diluted in 1% BSA and 0.3% Triton™ X-100 in PBS. Slides were incubated with primary antibodies overnight at 4 °C. For the MBP and NF double staining, the sections were processed with secondary antibody: anti-goat IgG-R.T.U. Dylight 594 (Vector Lab, DI-3794) for 1 h at RT to detect MBP or anti-rabbit IgG conjugated to biotin (dilution 1:200, Vector Lab, BA-1100) for 1 h at RT followed by incubation with Streptavidin-Alexa Fluor^®^ 488 to detect NF. The sections were mounted with VECTASHIELD HardSet Antifade Mounting Medium with DAPI (Vector Laboratories Inc., H-1500). Images were taken using AxioObserver with ApoTome microscope (Zeiss).

#### MCF7 cells

To visualise CHOP expression in MCF7 cells, cells were plated into round cover slips in 12-well plates at 5 × 10^4^ cells per well and allowed to adhere overnight as it is described in “MCF7 cell culture and drug treatment” section. Next day, 2 h prior to drug treatment, the media was exchanged for fresh media and then cell were treated with equal volume of vehicle (0.1% DMSO final concentration), 2.5 or 25 μM DAMGO for 48 h. To monitor effect of the ISRIB on CHOP and Bax expression in MCF7, cells were incubated in the presence of 10 μM oxycodone, morphine or DAMGO with or without of 40 nM ISRIB for 24 h. To prepare cover slips with cells for staining, cells were fixed in 4% warm formaldehyde (37 °C) for 10 min at RT and then permeabilized by incubation in 0.5% Triton™ X-100 for 3 min. After wash with PBS, cover slips were incubated with a solution [2% BSA, 0.3 M Glycine, 0.1% Tween^®^ 20 in BPS] for 1 h and then in blocking solution [2.5% horse serum, 0.1% Tween^®^ 20 in BPS] for another 1 h at RT in humidified chamber and then with primary antibodies against Bax (dilution 1:10,000; Proteintech Group, 50599-2-Ig) or CHOP (L63F7, dilution 1:400; Cell signaling, 2895) diluted in blocking solution for 2 h at RT in humidified chamber. After wash with TBS-T, cover slips were incubated with goat anti-mouse secondary antibodies conjugated to Alexa-594 (diluted 1:400; Abcam, ab150120) to detect CHOP or with goat anti-rabbit antibodies conjugated to Alexa-488 (diluted 1:400; Abcam, ab150077) to detect Bax diluted in blocking solution for 1 h at RT in humidified chamber. After wash with TBS-T and PBS, 10 μl drop of VECTASHIELD HardSet Antifade Mounting Medium with DAPI (Vector Laboratories Inc., H-1500) placed on each cover slips, which then been placed on a glass slides. Analysis of CHOP and Bax expression in MCF7 cells treated with opiates with or without ISRIB was performed three times using different cell culture for each experiment. Images were taken using 63X objective on an AxioObserver with ApoTome microscope (Zeiss). All acquisitions were performed using similar setup. An analysis of CHOP and Bax staining intensity was performed in ten separate fields of each slide containing 40–60 cells using AxioObserver software and repeated for all three experiments (120–200 total number of cells for each treatment). Mean intensity of CHOP and Bax staining in cells incubated in the presence of opiates with or without ISRIB was normalized to the mean intensity of CHOP and Bax staining in cells treated with buffer in the same experiment, set as one. Statistical significance was determined with a Student’s t-test, and data with p value lower than 0.05 was considered to be statistically different. Omission of the primary antibodies did not produce any fluorescent labelling.

### MTT assay

Cell viability was determined by the MTT bromide assay. The MCF7 cells (4 × 10^3^) were seeded in 96-well plates and incubated overnight. The next day, cells were treated with buffer (0.1% DMSO final concentration), 2.5 µM or 25 µM DAMGO for 48 h. At the end of this incubation, 20 μl of 12 mM MTT (Sigma, St.Louis, MO) was added to each well and incubated for another 1 h at 37 °C and the absorbance was determined at 570 and 630 nm using Victor3 1420 (Perkin-Elmer). Data from three different cell cultures were expressed as a delta (A_570_ − A_630_). Statistical significance of the difference between data on the presence of DAMGO versus buffer was determined with a Student’s t-test, and data with p value lower than 0.05 was considered to be statistically different.

## Results

### Prolonged oxycodone administration affects axonal brain structures

**Fig. 1:**
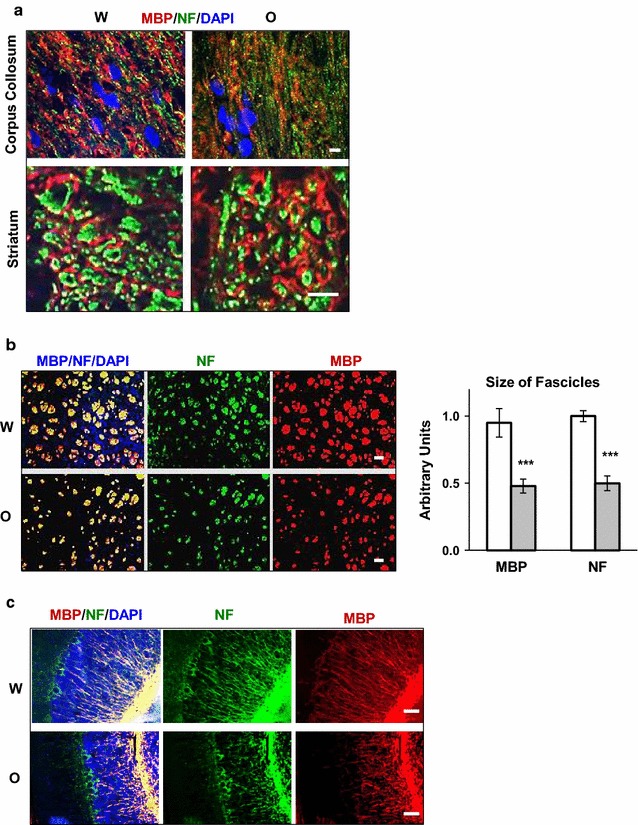
Prolong oxycodone administration affects axonal structures in rat brain. **a** Immunofluorescent analysis of MBP (red) and NF (green) in corpus callosum (upper images) and in striatum (lower images) of rats treated with water (W) or oxycodone (O). Blue is DAPI staying. Scale bars denote 5 μm for all images. Images represent immunofluorescent analysis of striatum and corpus callosum of three rats for each treatment from the different matching litters. **b** Immunofluorescent analysis of MBP (red) and NF (green) in axonal fascicles in striatum of rats treated with water (W) or oxycodone (O). Scale bar denotes 50 μm for all images. On right, graphs representing mean size of fascicles stained with MBP or NF antibodies in striatum. Open bars—water samples; grey filled bars—oxycodone samples. Data is expressed in arbitrary units normalized to that in water brain samples set as 1 (± SEM). Three to five brain slides containing whole striatum areas from three water-and three oxycodone-treated rats from the different matching litters were analysed. MBP, p < 0.001; NF, p < 0.001. **c** Immunofluorescent analysis of MBP (red) and NF (green) in cerebellum of rats treated with water (W) or oxycodone (O). Blue is DAPI staining. Scale bar denotes 50 μm for all images. Images represent immunofluorescent analysis of cerebellum areas of three rats for each treatment from the different matching litters

To investigate whether 30-days of 15 mg/kg oxycodone administration causes structural abnormalities in rat brain we analyzed staining for the myelin basic protein (MBP) and neurofilaments (NF) in various brain areas. Immunofluorescent analysis indicated morphological changes of both MBP and NF structures in the oxycodone-exposed brains compared to that in control animals. In the corpus callosum of water-treated rats, most of the NF fibers are surrounded by MBP (Fig. 1a, top left image). In contrast, in oxycodone-treated animals, significant numbers of NF fibers did not co-localize with MBP suggesting an excess of demyelinated axons in oxycodone-exposed corpus callosum (top right image). In the striatum of oxycodone-exposed rats, some axonal fascicles contained MBP shells with reduced NF staining or even without NF staining inside of them (Fig. 1a, lower images). Moreover, the average size of axonal fascicles stained with antibodies against either MBP or NF reduced more than twofold in oxycodone-exposed striatum (Fig. 1b). In the cerebellum of oxycodone-treated animals, some of the MBP-and NF-positive axons had weaker staining and did not extend to Purkinjie cells (Fig. 1c) suggesting axonal retraction or damage. These data suggest that prolonged oxycodone administration is linked to structural changes in white mater accompanied by a loss of MBP and NF.

### Oxycodone induces axonal damage

**Fig. 2:**
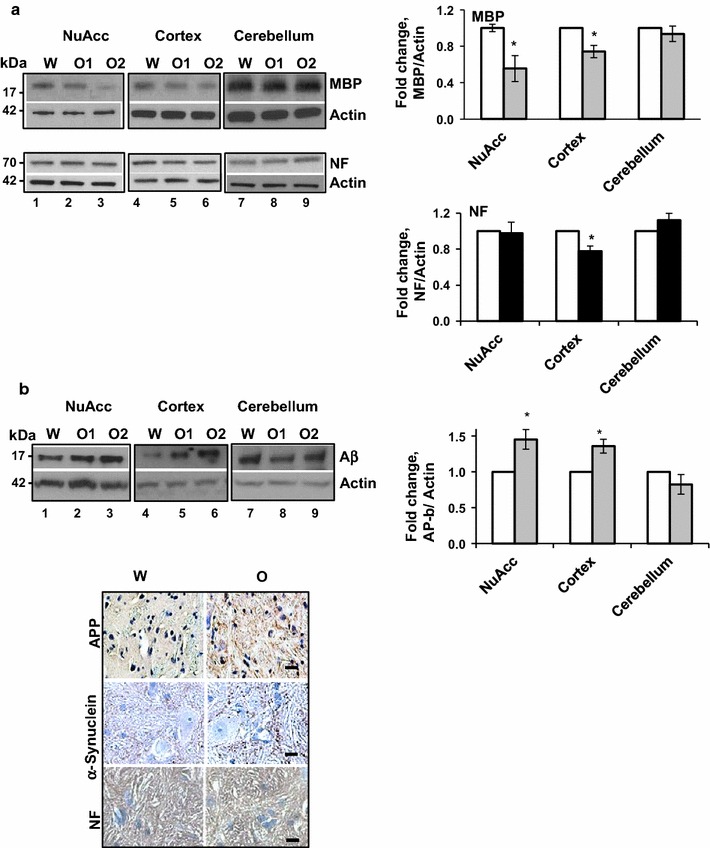
Chronic oxycodone induces axonal damage. **a** Western blot analysis of MBP and NF in rat brain lysates. On left, representative images of western blots of MBP, NF and actin in nucleus accumbens, cortex, and cerebellum of rats treated with water (W) or oxycodone (O1, O2). On right, graphs of the densitometric analysis of MBP and NF western blots. The graphs represent the mean ratio of signal of MBP to actin and NF to actin in the corresponding samples. Oxycodone data were normalized to water samples in corresponding tissues set as 1 (± SEM, n = 4). MBP analysis: NuAcc, p = 0.02; Cortex, p = 0.04; Cerebellum, p = 0.48. NF analysis: NuAcc, p = 0.9; Cortex, p = 0.02; Cerebellum, p = 0.22. Open bars—water samples; grey filled bars—MBP in oxycodone samples; black filled bars—NF in oxycodone samples. **b** Analysis of β-APP in rat brain lysates. Upper left panel, representative images of western blots of 17 kDa β-APP fragment and actin in nucleus accumbens, cortex, and cerebellum of rats treated with water (W) or oxycodone (O1, O2). Upper right panel, graphs of the densitometric analysis of β-APP western blots. The graphs represent the mean ratio of signal of β-APP to actin. Oxycodone data were normalized to water samples in corresponding tissues set as 1 (± SEM, n = 5). NuAcc, p < 0.03; Cortex, p < 0.05; Cerebellum, p = 0.26. Open bars—water samples; grey filled bars—oxycodone samples. Lower panel, representative images of immunohistochemical analysis of total APP, α-Synuclein, and NF in brain stem of rats treated with water (W) or oxycodone (O). Scale bar denotes 20 μm for all images

To investigate whether chronic oxycodone administration causes demyelination and loss of neurofilaments we monitored the level of myelin basic protein and neurofilaments in brain lysates from water and oxycodone-exposed animals. Western blot analysis showed significant decrease in MBP level in lysates from the nucleus accumbens and cortex but not from cerebellum of oxycodone exposed animals (Fig. 2a, upper images and graph on right). Interestingly, the level of neurofilaments significantly decreased only in lysate containing cortex but not nucleus accumbens or cerebellum tissues of oxycodone treated rats (Fig. 2a, lower images and graph on right).

To investigate whether prolonged oxycodone administration induces axonal damage we monitored the level of the lower molecular weight peptide of the beta-Amyloid precursor protein (β-APP). The accumulation of the β-APP has been shown to serve as an early marker of axonal damage after traumatic brain injury and in a variety of CNS diseases [33–35]. We have used the 6E10 antibodies that recognize both precursor APP and the toxic cleavage product, amyloid-β peptide (Aβ). Western blot analysis showed a slight increase in the level of the 17-kDa amyloid-β fragment in nucleus accumbens and cortex lysates from the oxycodone-exposed brain tissues (Fig. 2b, upper panel and graph on right). In addition, in brain stem of the drug-exposed rats, we observed dramatic accumulation of amyloid proteins along neurofilaments suggesting initiation of axonal damage after chronic oxycodone exposure (Fig. 2b, lower panel). Recently it was shown that accumulation of insoluble α-synuclein deposits is associated with oxidative stress and may lead to axonal degeneration [36]. We have observed significant accumulation of α-synuclein deposits in brain stem (Fig. 2b, lower panel) suggesting axonal pathologies typical for axonal degeneration.

### Chronic oxycodone activates astrocytes and increases percentage of TUNEL-positive cells in rat brain

**Fig. 3:**
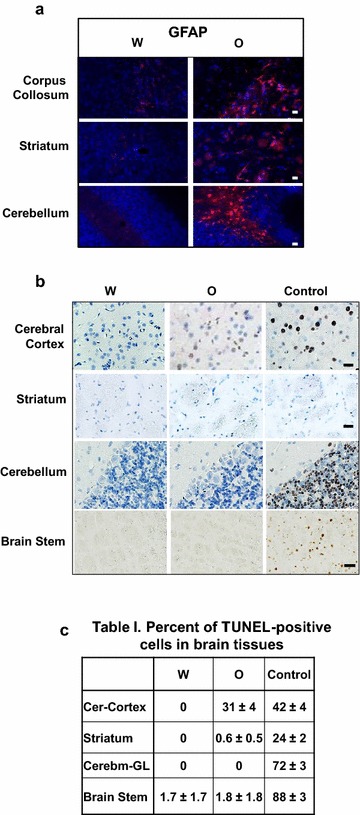
Chronic oxycodone activates astrocytes and increases percentage of TUNEL-positive cells in rat brain. **a** Immunofluorescent analysis of GFAP (red) in corpus callosum, striatum, and cerebellum of rats treated with water (W) or oxycodone (O). Scale bar denotes 20 μm for all images. **b** TUNEL assay of cells in cerebral cortex, striatum, cerebellum, and brain stem areas of rats treated with water (W) or oxycodone (O). Control—slides with oxycodone tissues were pretreated with TACS-Nuclease solution™ to serve as a positive control. Scale bar denotes 50 µm for brain stem images and 20 µm for all other images. **c** Table I. Percent of TUNEL-positive cells in rat brain tissues exposed to water (W) oxycodone (O) and in control slides. Data presented as mean value of TUNEL-positive cells (± SEM). Cerebral cortex: three separate fields containing 33–91 cells per field, oxycodone versus water p < 0.01, and oxycodone versus control p < 0.001. Striatum: three separate fields containing 53–76 cells per filed, oxycodone versus water p = 0.4, and oxycodone versus control p = 2×10^−4^. Cerebellum, granular layer: three separate fields containing 85–229 cells per field, oxycodone versus control p < 0.001. Brain stem: four separate fields containing 38–63 cells per field, oxycodone versus control p < 0.001

White matter pathologies promote infiltration of reactive astroglia to clear the axonal and myelin debris (reviewed in [37]). Astrocytes become reactivated following CNS injury leading to astrogliosis. This process is characterized by astroglial hypertrophy with concomitant rapid synthesis of GFAP (reviewed in [38]). It was suggested that increased GFAP level may serve as a marker of axonal damage in patients with chronic neuropathies [39] and with traumatic brain injuries [40]. We observed modest increase of the GFAP-positive astrocytes in corpus callosum, striatum and cerebellum of oxycodone-exposed animals (Fig. 3a).

**Fig. 4:**
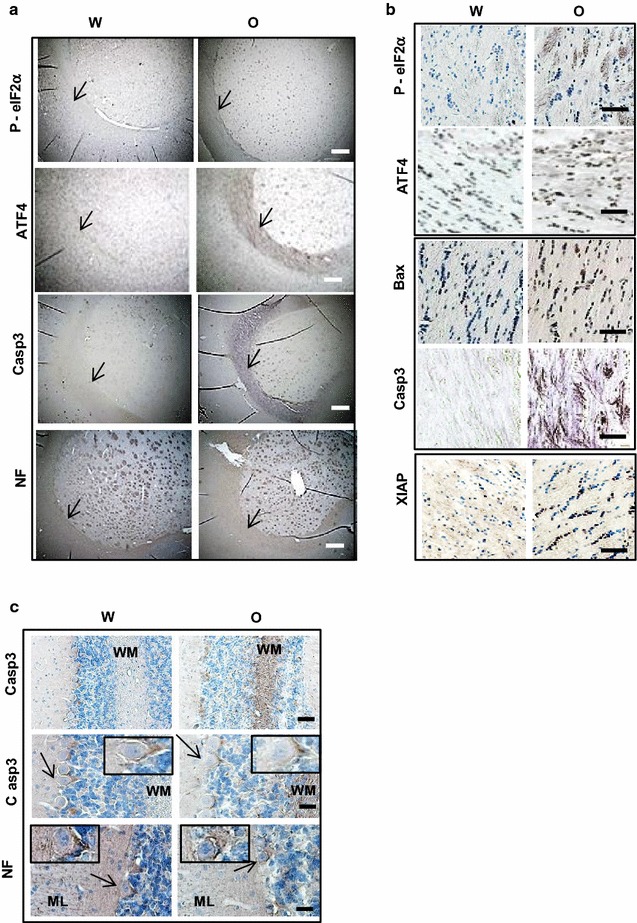
Oxycodone activates pro-apoptotic machinery in white matter. **a** Immunohistochemical analysis of phospho-eIF2α, ATF4, cleaved caspase 3 (Casp3), and NF staining in cortex area containing corpus callosum of rats treated with water (W) or oxycodone (O). Arrows point to corpus callosum. Scale bar denotes 200 μm for all images. **b** Immunohistochemical analysis of phospho-eIF2α, ATF4, Bax, cleaved caspase 3, and XIAP staining in corpus callosum of rats treated with water (W) or oxycodone (O). Please note that Casp3-slides were not counterstained with Hematoxyllin. Scale bar denotes 50 μm for all images. **c** Immunohistochemical analysis of cleaved caspase 3 and NF staining in cerebellum of rats treated with water (W) or oxycodone (O). Arrows point to Purkinjie cells enlarged in the inserts. Scale bar denotes 50 μm for the top Casp3 images and 20 μm for the middle Casp3 and lower NF images

To investigate whether prolonged oxycodone administration induces neuronal cell death we performed the TUNEL (*T*erminal deoxynucleotidyl transferase-mediated d*U*TP *n*ick *e*nd *l*abeling) assay that detects fragmented DNA in cells undergoing to apoptosis (Fig. 4a). As a positive control, we pre-treated a separate slide containing oxycodone-exposed brain tissue with DNase followed by the TUNEL assay (Fig. 3b, Control). We observed a moderate increase in the percentage of TUNEL-positive cells in the cerebral cortex of the oxycodone-exposed animals compared to that in water-exposed tissues (Fig. 3c). However, the intensity of staining in oxycodone tissues was much lower compared to that in the control slides suggesting low DNA fragmentation level in oxycodone-exposed cerebral cortex (Fig. 3b). Interestingly, in brain areas containing striatum, cerebellum and brain stem, there were no detectable TUNEL-positive cells in both, water and oxycodone brain slices (Fig. 3b, c). These data suggests that prolonged oxycodone administration causes slight induction of DNA fragmentation in cells located in cerebral cortex but not in striatum, cerebellum or brain stem.

### Chronic oxycodone activates pro-apoptotic machinery in white matter

**Fig. 5:**
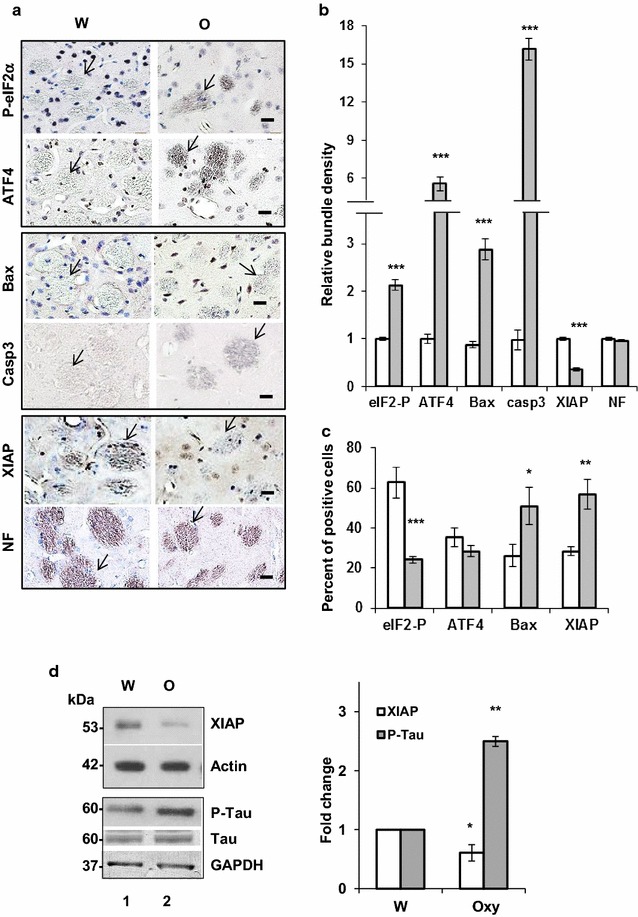
Oxycodone differentially induces pro-and anti-apoptotic signaling in cell bodies and axons. **a** Immunohistochemical analysis of phospho-eIF2α, ATF4, Bax, cleaved caspase 3 (Casp3), XIAP, and NF in striatum of rats treated with water (W) or oxycodone (O). Arrows point to axonal fascicles. Scale bar denotes 20 μm for all images. **b** Graphs of the densitometric analysis of phospho-eIF2α, ATF4, Bax, cleaved caspase 3 (Casp3), XIAP, and NF staining in bundles of the striatum rats treated with water (open bars) or oxycodone (grey filled bars). The graphs represent relative density measured as the ratio of the mean values of intensities obtained from oxycodone exposed tissue to the bundle intensities in striatum of water-exposed rat brains set as 1 (± SEM). Data obtained from three animals for each treatment. Statistical analysis was performed using Student’s t-test. eIF2α-P, p < 0.001; ATF4, p < 0.001; Bax, p < 0.001; cleaved caspase 3, p < 0.001; XIAP, p < 0.001; and NF, p = 0.3. **c** Graphs of the densitometric analysis of phospho-eIF2α, ATF4, Bax, cleaved caspase 3 (Casp3), XIAP, and NF staining in cell bodies in striatum of rats treated with water (open bars) or oxycodone (grey filled bars). Data presented as a percent of positively stained cells relative to total number of cells. Data obtained from three animals for each treatment, Data expressed as mean value (± SEM). Statistical analysis of positively stained cells in oxycodone relative to water exposed striatum was performed using Student’s t-test. Phospho-eIF2α, p < 0.001; ATF4, p = 0.2; Bax, p < 0.05; and XIAP, p < 0.01. **d** Western blot analysis of XIAP and phospho(Thr181)-Tau in rat cortex lysates. Left panel, the representative images of western blots of XIAP, phospho-Tau, total Tau, actin and GAPDH in cortex of rats treated with water (W) or oxycodone (O). Right panel, graphs of the densitometric analysis of XIAP and phospho-Tau western blots. The graphs represent the mean ratio of signal of XIAP to actin and phospho-Tau to total Tau. Oxycodone data were normalized to water samples in corresponding tissues set as 1 (± SEM). XIAP, n = 3, p < 0.05; P-Tau, n = 4, p < 0.003. Open bars—XIAP; and grey filled bars—P-Tau

Recently, we have shown that chronic oxycodone exposure leads to activation of the integrated stress response (ISR) in rat brain [25]. Activation of the ISR helps cells to cope with stress and promotes their survival [30]. However, prolonged stimulation of the ISR may lead to activation of the pro-apoptotic machinery and neuronal death [41–43].To investigate whether chronic oxycodone exposure activates pro-apoptotic signaling we monitored expression of activated caspase 3 and Bax and also induction of the ISR in rat brain. Immunohistochemical analysis showed significant increase of phospho-eIF2α and ATF4 in corpus callosum (Fig. 4a, b), cerebellum [25], and striatum (Fig. 5a) confirming activation of the ISR in the brains of oxycodone-exposed rats. We also observed increase in Bax and activated caspase 3 staining in corpus callosum (Fig. 4a, b), cerebellum (Fig. 4c), and striatum (Fig. 5a) of oxycodone-treated animals. Interestingly, cellular localization of activated caspase 3 was different from localization of phospho-eIF2α, ATF4 and Bax. Please note that slides stained for activated caspase 3 were not counterstained with Hematoxyllin to enable detection of caspase 3-specific staining in cell bodies. In all oxycodone-exposed brain samples activated caspase 3 was predominantly detected along axons. In corpus callosum, cleaved caspase 3 was absent in water-treated samples but showed strong staining in axonal tracks of oxycodone-treated samples (Fig. 4b). In cerebellum of oxycodone-exposed rats, activated caspase 3 localized in white matter and in fibers surrounding Purkinjie cell bodies in granular layer resembling the neurofilament staining (Fig. 4c) confirming caspase 3 presence in axons. Similarly, in striatum, activated caspase 3 was detected predominantly in axonal fascicles (Fig. 5a). Statistical analysis showed more than threefold increase in staining of activated caspase 3 in corpus callosum, more than fivefold increase in anterior commissure and about a sevenfold increase in cerebellum white matter of oxycodone-treated animals (data not shown). In contrast, phospho-eIF2α, ATF4 and Bax were detected in both cell bodies and axonal fibers in corpus callosum of oxycodone exposed animals (Fig. 4b), cerebellum (data not shown), and striatum (Fig. 5a).

The ISR also regulates expression of the X-linked inhibitor of apoptosis, XIAP, via translational inhibition by phopsho-eIF2α and degradation by the ATF4-dependent ligase [44]. We investigated whether chronic oxycodone exposure affects XIAP expression. Western blot analysis of total lysate prepared from cortex showed significant decrease in overall XIAP level in oxycodone treated rats suggesting activation of pro-apoptotic pathways (Fig. 5d). In addition, in oxycodone cortex lysates, we observed about 2.5-fold increase in phospho(Thr181)-tau, one of the biomarkers of neuronal degeneration (reviewed in [45]) (Fig. 5d). Detailed analysis of cellular localization of XIAP revealed that in the striatum of oxycodone exposed animals, the level of XIAP decreased in axonal bundles that coincided with increase in phospho-eIF2α, ATF4, Bax and activated caspase 3 (Fig. 5a, b) suggesting activation pro-apoptotic signaling in axons. In contrast, in cell bodies in oxycodone-exposed striatum, XIAP signal increased while the level of phospho-eIF2α decreased (Fig. 5a, c). Similar distribution was observed in corpus callosum (Figs. 4b). An increase in XIAP level and lack of activated caspase 3 in cell bodies suggests activation of pro-survival pathways in soma of neurons by chronic oxycodone exposure. These results suggest that the ISR and pro-apoptotic machinery may be differentially activated by chronic oxycodone depending on their compartmentalisation in neuronal cells.

### Opioids activate pro-apoptotic signaling in MCF7 cells

**Fig. 6:**
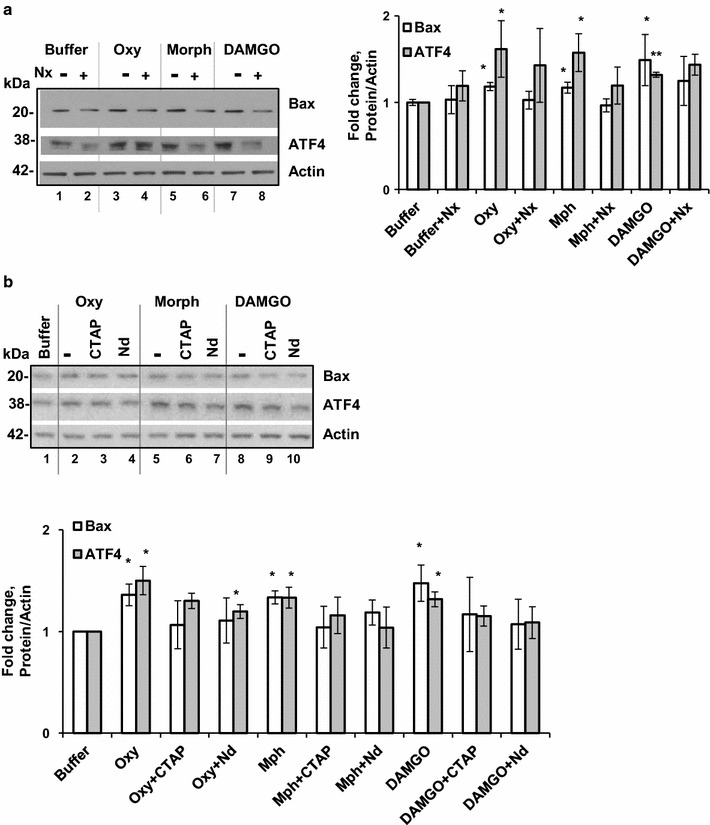
Opioids activate pro-apoptotic signaling in MCF7 cells. **a** Western blot analysis of Bax and ATF4 in MCF7 cells treated with opioids and naloxone. Left panel, representative images of western blots of Bax, ATF4 and actin in MCF7 cells treated with buffer, 10 μM oxycodone (Oxy), 10 μM morphine (Morph), or 10 μM DAMGO in the presence (+) or absence (−) of 10 μM naloxone (Nx) for 24 h. Right panel, graphs of the densitometric analysis of Bax and ATF4. The graphs represent the mean ratio of signal of Bax and ATF4 to actin. Open bars—Bax; grey filled bars—ATF4. All data were normalized to buffer samples set as 1 (± SEM, n = 3). ATF4: oxycodone versus buffer, p < 0.02; morphine versus buffer, p < 0.03; and DAMGO versus morphine, p < 0.01. Bax: oxycodone versus buffer, p < 0.02; morphine versus buffer, p < 0.05; and DAMGO versus buffer, p < 0.05. **b** Western blot analysis of ATF4 and Bax in MCF7 cells treated with opioids and CTAP or naltrindole. Upper panel, representative images of western blots of ATF4, Bax and Actin in MCF7 cells treated with buffer, 10 µM oxycodone, 10 µM morphine, or 10 µM DAMGO in the presence or absence of 0.3 µM CTAP or 0.3 µM naltrindole (Nd) for 24 h. Lower panel, graphs of the densitometric analysis of ATF4 and Bax western blots. The graphs represent the mean ratio of signal of ATF4 and Bax to actin. Open bars—Bax; grey filled bars—ATF4. All data were normalized to buffer samples set as 1 (± SEM, n = 3). ATF4: oxycodone versus buffer, p < 0.03; morphine versus buffer, p < 0.04; DAMGO versus buffer, p < 0.02; and oxycodone + Nd versus oxycodone, p < 0.05. Bax: oxycodone versus buffer, p < 0.03; morphine versus buffer, p < 0.02; and DAMGO versus buffer, p < 0.05

Previously we demonstrated that oxycodone activates the ISR in human breast adenocarcinoma MCF7 cells, which express μ-opioid (MOP) receptor [25]. To investigate whether other the MOP receptor agonists induce the ISR and pro-apoptotic signaling, we incubated MCF7 cells in the presence of 10 μM oxycodone, morphine, or opioid peptide DAMGO for 24 h and monitored expression levels of ATF4 and Bax. We did not observe any morphological changes in MCF7 cell after these treatments (data not shown). Western blot analysis revealed that similar to oxycodone, morphine and DAMGO stimulated ATF4 synthesis in MCF7 cells suggesting activation of the integrated stress response (Fig. 6a). All three compounds, oxycodone, morphine, and DAMGO also increased levels of Bax in MCF7 cells indicating stimulation of pro-apoptotic signaling. Pre-incubation of cells with pan-opioid antagonist naloxone partially prevented increase in ATF4 and Bax by oxycodone, morphine or DAMGO, but it was not statistically significant. Naloxone is a nonselective opioid receptor antagonist. To investigate the role of the MOP receptor in activation of the ISR and pro-apoptotic signaling we incubated MCF7 cells with oxycodone, morphine, or DAMGO in the presence of 0.3 µM CTAP, a MOP receptor selective antagonist [46]. Similarly to naloxone, CTAP partially prevented opioid-induced increase in ATF4 and Bax levels, but it was not statistically significant (Fig. 6b). We also investigated whether activation of the delta-opioid receptor by opioids triggers the ISR and pro-apoptotic signaling. We incubated MCF7 cells with oxycodone, morphine, or DAMGO in the presence of 0.3 µM naltrindole, a delta-opioid receptor selective antagonist [47]. However, similar to naloxone and CTAP, suppression of the opioid-induced increase in ATF4 and Bax levels by naltrindole was not statistically significant, except for the changes in the ATF4 levels in oxycodone samples. These data suggest that both opioid (mu-and delta-) and non-opioid-receptor signaling pathways may contribute to activation of the ISR and pro-apoptotic signaling in MCF7 cells by oxycodone, morphine, and DAMGO.

### Inhibition of the ISR reduces an opioid-induced activation of pro-apoptotic signaling in MCF7 cells

**Fig. 7:**
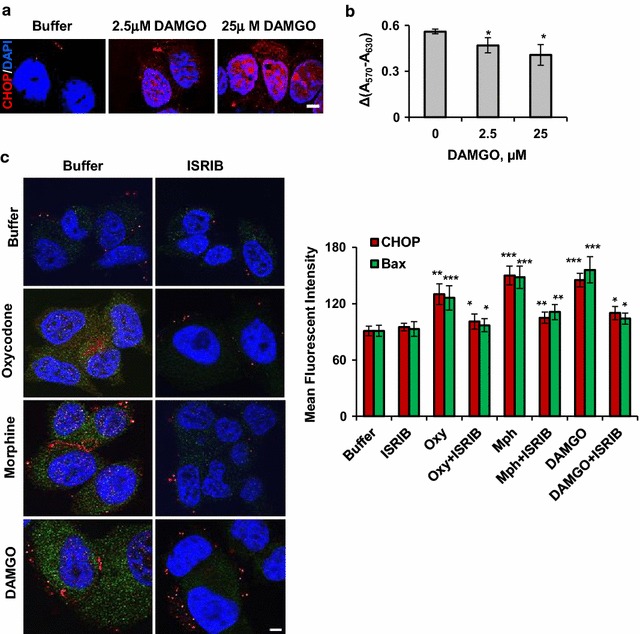
ISRIB suppresses opioid-induced increase in CHOP and Bax expression. **a** Immunofluorescent analysis of CHOP (red) expression in MCF7 cells incubated with buffer, 2.5 µM or 25 µM DAMGO for 48 h. Blue is DAPI staining. Scale bar denotes 5 µm for all images. 2.5 µM DAMGO versus buffer, p < 0.05; 25 µM DAMGO versus buffer, p < 0.02; **b** MTT analysis of cells growing in the presence or absence of 2.5 µM or 25 µM DAMGO for 48 h. Graph represents mean values of delta(A_570_ − A_630_) (± SEM, n = 3). **c** Left panel, representative immunofluorescent images of CHOP (red) and Bax (green) in MCF7 cells incubated with buffer, 10 µM oxycodone, morphine or DAMGO and with or without 40 nM ISRIB. Scale bar denotes 5 µm for all images. Right panel, graphs representing mean fluorescent intensity of CHOP and Bax signals in MCF7 cells similar to experiments shown on the left panel. Red bars—CHOP; green bars—Bax. All data were normalized to buffer samples set as 1 (± SEM, n = 3). CHOP: oxycodone, morphine and DAMGO versus buffer, p < 0.01, p < 0.001, p < 0.001 respectively; oxycodone plus ISRIB versus oxycodone, p < 0.03; morphine plus ISRIB versus morphine, p < 0.008; DAMGO plus ISRIB versus DAMGO, p < 0.02. Bax: oxycodone, morphine and DAMGO versus buffer, p < 0.001 for all opioids; oxycodone plus ISRIB versus oxycodone, p < 0.04; morphine plus ISRIB versus morphine, p < 0.01; DAMGO plus ISRIB versus DAMGO, p < 0.04

Prolong activation of the ISR was shown to stimulate expression of the C/EBP homologues protein, CHOP [48]. CHOP is a transcription factor that involved in stress response and neuronal apoptosis [42, 43, 49]. Incubation of MCF7 cells with 2.5 or 25 µM DAMGO for 48 h led to a dose-dependent increase in CHOP level (Fig. 7a) suggesting activation of the ATF4-CHOP axes by DAMGO. Moreover, 2.5 and 25 µM DAMGO treatment for 48 h inhibited metabolic activity in MCF7 cells in a dose-dependent manner as it was measured by the MTT assay (Fig. 7b). Together, increased level of CHOP and decreased MCF7 cell viability suggest induction of pro-apoptotic signaling by DAMGO in a dose-dependent manner.

To investigate whether opioid-induced pro-apoptotic signaling is linked to activation of the ISR, we treated MCF7 cells grown in the presence of 10 μM oxycodone, morphine or DAMGO with 40 nM ISRIB for 24 h (Fig. 7c). ISRIB is a small molecule that inhibits expression of ATF4 [50]. Immuno-fluorescent staining of MCF7 cells treated with opioids and ISRIB revealed that ISRIB efficiently suppressed an opiates-induced increase in CHOP and Bax expression compared to that in the presence of opioids alone (Fig. 7c). Altogether, these data indicate that opioid-induced integrated stress response contributes to activation of pro-apoptotic signaling in MCF7 cells.

## Discussion

There are several mechanisms suggested to contribute to opioid-induced degeneration of white matter. Demyelination due to diminish capacity of myelinating oligodendrocytes and/or due to astro-glia activated inflammation have been suggested as one of the triggers of white matter loss. MBP synthesised by mature oligodendrocytes in the CNS provides insulation to axons and it thought to protect them from the toxic environment and injury [51]. A recent study of brain pathologies in heroin-abusers identified not only disruption of white matter but also myelin impairment [11]. Another study showed activation of microglia in brains of opiate abusers [52]. It was suggested that apoptosis of oligodendrocytes, collapse and fracture of myelin sheath and microvascular damage [13] may be the reason of leukoencephalopathy development after heroin intoxication [8]. In our study, we observed that chronic oxycodone administration caused morphological changes in axonal tracks, accumulation of activated caspase 3 in white matter and increase in reactivated astrocytes in brain areas containing corpus callosum, striatum and cerebellum. The greatest effect by chronic oxycodone exposure was detected in striatum and nucleus accumbens characterized by reduction in axonal bundles, loss of MBP and NF but increase in APP-β suggesting axonal damage and loss. Our observations are in agreement with a study demonstrating correlation between the duration of prescription opioid exposure of patients and greater changers in white matter anisotropy of efferent and afferent pathways of the amygdala, especially connectivity between amygdala-inferior orbital frontal cortex and amygdala-nucleus accumbens [20]. The fact that we observed loss of MBP in the nucleus accumbens and cortical areas, while the level of neurofilaments decreased only in cortex areas suggests that neuronal demyelination might precede axonal loss or that loss of MBP and NF occur via different mechanisms.

Apoptosis of neuronal cells could be another mechanism contributing to the white matter loss. Chronic or excessive opioids were linked to activation of apoptosis in brains of morphine [24] or heroin addicted rats [21]. In our study, we observed the induction of DNA fragmentation in cells located in the cerebral cortex but not in the striatum, cerebellum or brain stem. Morphine was shown to induce overexpression of several pro-apoptotic proteins including Bax and activated caspases 3 in the spinal cord dorsal horn [23, 53], neonatal rat cortex and amygdala [54], and mouse neural progenitor cells [55] and death receptor Fas and caspase 3 in rat hippocampus [24]. In agreement with these studies, we demonstrated that prolonged administration of oxycodone increases the level of activated caspase 3 and expression of pro-apoptotic Bax in rat brain suggesting induction of apoptosis. However, we observed modest induction of DNA damage in cerebellar cortex but not in striatum, cerebellum and brain stem in oxycodone exposed rat brains arguing against induction of apoptosis in neuronal cells. Interestingly, activated caspase 3 was predominantly detected in axonal tracks but not in cell bodies in brain slices of oxycodone treated animals. In contrast, the level of XIAP, a protein that was shown to control caspase 3 activity in degenerating axons [56], increased in cell bodies and decreased in axons after oxycodone exposure.

Robust accumulation of activated caspase 3 in white matter: corpus callosum, anterior commissure, axonal fascicles in striatum, and cerebellar white matter, amid low or no detectable apoptosis suggests that chronic oxycodone exposure triggers caspase proteolytic activity in an axon-localized manner that may lead to axonal damages, but preserves cell bodies. Such strategy will allow cells to survive and restore their function after stress ceases. Recently it was shown that in mature neurons, caspases perform an important function in modulating axonal pruning and synapse elimination without induction of neuronal death (reviewed in [57]). Thus, activation of caspase 3 in axons after chronic oxycodone exposure may indicate an increased rate of axonal pruning leading to loss of white matter integrity. We did not notice a difference in density or width of white matter in corpus callosum, anterior commissure, or cerebellum between water and oxycodone treated rats. However, in the cerebellum of oxycodone exposed animals we observed deformation of axonal tracks. Also, in oxycodone-exposed striatum, the average size of axonal fascicles was more than two-fold smaller than in water treated animals suggesting axonal damage by prolonged oxycodone administration. In agreement with that, we observed increase in phospho-tau, an indicator of destabilization of axonal microtubules, and also increased level of amyloid precursor protein β-APP in oxycodone-exposed brains, which serves as an early biomarker of axonal damage after traumatic brain injury and in a variety of CNS diseases [33–35]. Our data are in agreement with other animal studies that demonstrated a decrease in neurofilaments in the rat frontal cortex [58] but increased expression of the β-APP in rats Locus Ceruleus after chronic morphine treatment [59]. Investigation of opiate-abusers brains also demonstrated accumulation of β-APP and hyper-phosphorylated tau in various brain regions [52, 60].

What could be the mechanism of the opioid-induced axonal degeneration? One of the mechanisms contributing to a deleterious effect of chronic opioids on axonal integrity is impaired axonal transport due to loss of neurofilaments, destabilization of microtubules and accumulation of β-APP. It was demonstrated that chronic morphine administration resulted in reduction of neurofilaments in ventral tegmental area (VTA) and was associated with impaired axonal transport from VTA to nucleus accumbens by 50% in rat brain [61]. In addition, stress-altered local translation may contribute to impairment of axonal transport (reviewed in [62]). It was demonstrated that axonal application of oligomeric Aβ_1–42_ induces axonal translation of ATF4 mRNA. Moreover, expression of ATF4 promoted retrograde neurodegenerative signals in rat neurons [63]. Thus, even it is tempting to speculate that in neuronal cells there is a specific mechanism protecting cell bodies at the expense of axons, retrograde spread of neurodegenerative signals may eventually lead to a neuronal death.

ATF4 is a critical member of the integrated stress response signaling. In our previous study we demonstrated that chronic oxycodone administration is linked to activation of the integrated stress response (ISR) in rat brains [25]. The integrated stress response is a general mechanism that allows cells to adapt to various types of stresses [30]. The main event of the ISR, phosphorylation of initiation translation factor eIF2α at Ser-51, leads to attenuation of global protein synthesis but stimulation of translation of specific mRNA with upstream open reading frames such as ATF4 mRNA [27]. The ATF4 is a transcription factor that induces expression of genes encoding chaperones, redox and proteasomal proteins [28]. Inhibition of global translation allows cells to reduce the load of misfolded proteins in the endoplasmic reticulum (ER). At the same time, expression of the ATF4-regulated genes promotes synthesis of the proteins necessary for survival during stress and recovery after stress ceases [64]. Under severe or prolonged stress, ATF4 also induces expression of the growth arrest and DNA damage/C/EBP homology protein, GADD153/CHOP mRNA [27, 65], which is then translationally activated by phosphorylation of eIF2α [48, 64]. CHOP is a transcription factor that activates several target genes involved in cell growth, regeneration, differentiation and apoptosis [49, 66, 67]. It was shown that prolonged activation of the ISR may lead to activation of apoptosis via two separate routes: through increased expression of pro-apoptotic CHOP and through reduced levels of anti-apoptotic XIAP. It was shown that ATF4-CHOP axis transcriptionally stimulates expression of p53 upregulated modulator of apoptosis, PUMA, during ER stress and induces apoptosis in neurons [43] via activation of pro-apoptotic machinery including Bax and caspases [68]. Second, the ISR regulated pro-apoptotic axis involves reduction of the X-linked inhibitor of apoptosis, XIAP, via translational inhibition by phopsho-eIF2α and degradation by the ATF4-dependent ligase [44]. In degenerating axons, XIAP was shown to prevent apoptosis by binding to caspases and suppressing their proteolytic activity [56]. In our study we showed that in oxycodone-exposed brain areas containing white matter, ATF4 and phospho-eIF2α co-localized with Bax and cleaved caspase 3. On the other hand, induction of the ISR may also reduce the level of anti-apoptotic protein XIAP: phosphorylation of eIF2α was shown to inhibit translation of XIAP mRNA, and ATF4 induces XIAP degradation via ubiquitination [44]. In our study we observed that XIAP and phospho-eIF2α levels in cell bodies and axonal tracks changed in opposite directions after chronic oxycodone exposure. The percentage of cells with phospho-eIF2α dropped almost threefold while percentage of cells expressing XIAP increased more than twofold in oxycodone striatum compared to that in water treated animals. In contrast, in axonal fascicles, staining for phospho-eIF2α increased while XIAP level decreased after prolonged oxycodone exposure. Thus, it is possible that the oxycodone-triggered integrated stress response affects local translation in axons leading to decreased level of XIAP that promotes caspase 3 proteolytic activation and, thus, axonal degeneration. However, in cell bodies, chronic oxycodone exposure may activate pro-survival signaling via suppression of the ISR (reduced phosphorylation of eIF2α) and increased expression of XIAP.

Using breast adenocarcinoma MCF7 cells that are known to express MOP receptor, we showed that, in addition to oxycodone, morphine and DAMGO also induce the ISR and increase the Bax level. Moreover, treatment of cells with the ISR inhibitor, ISRIB, prevented opioid-stimulated increase in CHOP and Bax expression, indicating the contribution of the ISR to opioid-induced cellular toxicity. Our data suggest that both opioid and non-opioid pathways are responsible for induction of the ISR and pro-apoptotic signaling by oxycodone, morphine, and DAMGO. MCF7 cells express all three types of opioid receptors: they have a high number of binding sites for the MOP and kappa-opioid receptors and lower affinity for the delta-opioid receptor [69]. In our study, we observed that a selective MOP receptor antagonist (CTAP) and a selective delta-opioid receptor antagonist (naltrindole) partially suppressed opioid-induced ISR and pro-apoptotic signaling, suggesting contribution of the mu-and delta-opioid receptors to opioid-induced cellular toxicity. It is also possible that a kappa-opioid receptor may be involved in the induction of the ISR and pro-apoptotic signaling by chronic opioids. However, this suggestion is challenged by the fact that naloxone, a pan-opioid receptor antagonist, failed to inhibit opiate-induced pro-apoptotic signaling. A second possibility is that chronic opioids induce cell apoptosis by targeting non-opioid receptors and/or signaling pathways. MCF7 cells were shown to express functional N-methyl-D-aspartate receptor (NMDAR) [70], and this signaling was shown to contribute to morphine-induce neurotoxicity in a rat model [23]. Also, it was shown that opioids inhibit MCF7 cell growth in an estrogen-dependent manner [69]. In addition, MCF7 cells express the opioid-growth factor receptor (OGFr) [71], which contributes to morphine-induced suppression of lung cancer cell proliferation [72]. In another study, induction of pro-apoptotic death in SH-SY5Y cells by morphine was opioid-receptor independent and occurred via JNK-ROS-mitochondria-dependent apoptosis signaling [73]. Thus, it is possible that a high dose of opioids may induce the ISR and pro-apoptotic signaling in MCF7 cells via a combination of opioid and non-opioid receptor routes.

Reduction of brain white matter has also been demonstrated in alcohol and other drug addicts: alcohol abuse [74], alcohol and drug abuse [75, 76], cannabinoids [77, 78]. Also, methamphetamine [79, 80] and cocaine were shown to induce degeneration of myelinated fibers and axons [81]. Thus, it is possible that the opioid-induced leukoencephalopathy may have a common mechanism with other drugs of abuse—locally (in axons) activated integrated stress response. Indeed, increase in ATF4 level was demonstrated in response to stress and amphetamine administration in rats [82], suggesting the ISR as a common rout of neuronal degeneration by the drugs of abuse. It was suggested that ATF4 serves as a pro-death transcription factor sensitizing neurons to oxidative stress [83]. In agreement with that, knockdown of axonal ATF4 mRNA prevented neuronal loss in an Aβ_1–42_-induced neurodegeneration model [63]. Our data suggest that the ISR may serve as a therapeutic target to protect against development of drug-induced brain pathology and restore brain connectivity lost after drug-abuse. In agreement with that, pharmacological suppression of ATF4 expression with ISRIB was shown to improve memory formation in rats [50] and to provide neuroprotection in prion-diseased mice [84]. In other degenerative models, dietary suppression of the ATF4 pathway alleviated age-associated neuronal pathology [85] and muscle degeneration [86] in rats.

## Conclusions

Our data suggest that chronic opioid administration may cause axonal degeneration via demyelination due to loss of MBP or induction of degenerative processes in axons. In addition, our data also suggest that opioids can induce axonal degeneration via the ISR regulated mechanisms: alternation in axonal translation, stimulation of the caspase-induced proteolytic activity, degradation of the axonal skeleton and alternation in axonal transport.

## Abbreviations

APP: amyloid precursor protein
ATF4: cAMP element binding transcription factor 4 or activating transcription factor 4
BSA: bovine serum albumin
CHOP: DNA damage/C/EBP homology protein
CTAP: _D_-Phe-Cys-Tyr-_D_-Trp-Arg-Thr-Pen-Thr-NH_2_
DAMGO: [_D_-Ala^2^, *N*-MePhe^4^,Gly-ol]-enkephalin
CNS: central nervous system
eIF2α: eukaryotic initiation factor 2 alpha
ER: endoplasmic reticulum
Fisher’s PLSD: Fisher’s protected least significant difference
ISR: integrated stress response
ISRIB: integrated stress response inhibitor
GAPDH: glyceraldehyde 3-phosphate dehydrogenase
MBP: myelin basic protein
MOP: µ-opioid
NF: neurofilaments
OGFr: opioid-growth factor receptor
Oxy: oxycodone
PUMA: p53 upregulated modulator of apoptosis
RT: room temperature
uORF: upstream open reading frame
UPR: unfolded protein response
UTR: untranslated region
WM: white matter
XIAP: X-linked inhibitor of apoptosis
